# Comparing the case-mix of frail older people at home and of those being admitted into residential care: a longitudinal study

**DOI:** 10.1186/s12877-020-01593-w

**Published:** 2020-06-05

**Authors:** Johanna de Almeida Mello, Sophie Cès, Dirk Vanneste, Thérèse Van Durme, Chantal Van Audenhove, Jean Macq, Brant Fries, Anja Declercq

**Affiliations:** 1 LUCAS, Center for Care Research and Consultancy, KULeuven, Leuven, Belgium; 2 Institute of Health and Society, Université Catholique de Louvain, Brussels, Belgium; 3 School of Public Health, University of Michigan, Ann Arbor, MI USA; 4 CeSO: Centre for Sociological Researc, KULeuven, Leuven, Belgium

**Keywords:** Case-mix, Health services optimization, Home care, Long-term care, Resource utilization, RUG-III/HC

## Abstract

**Background:**

In order to optimize interventions and services in the community, it is important to identify the profile of persons who are able to stay at home and of those who are being admitted into residential care. Understanding their needs and their use of resources is essential. The main objective of the study is to identify persons who are likely to enter residential care based upon their needs and resource utilization, so that care providers can plan interventions effectively and optimize services and resources to meet the persons’ needs.

**Methods:**

This is a longitudinal quasi-experimental study. The data consists of primary data from the community setting collected every six months during the period of 2010–2016. Interventions had the goal of keeping older people longer at home. Participants were at least 65 years old and were living in the community. The interRAI Resource Utilization Group system (RUG-III) was used to calculate the case-mix indexes (CMI) of all participants. Comparisons were made between the case-mix of those who were still living at home and those who were admitted into residential care at follow-up.

**Results:**

A total of 10,289 older persons participated in the study (81.2 ± 7.1 yrs., 69.1% female). From this population, 853 participants (8.3%) were admitted into residential care. The CMI of the persons receiving night care at home were the highest (1.6 at baseline and 1.7 at the entry point of residential care), followed by persons receiving occupational therapy (1.5 at baseline and 1.6 at the entry point of residential care) and persons enrolled in case management interventions with rehabilitation (1.4 at baseline and 1.6 at the entry point of residential care). The CMIs at follow-up were significantly higher than at baseline and the linear regression model showed that admission to residential care was a significant factor in the model.

**Conclusions:**

The study showed that the RUG-III system offers possibilities for identifying persons at risk of institutionalization. Interventions designed to avoid early nursing home admission can make use of the RUG-III system to optimize care planning and the allocation of services and resources. Based on the RUG-III case-mix, resources can be allocated to keep older persons at home longer, bearing in mind the complexity of care and the availability of services in the community.

## Background

Staying at home for as long as possible can be a suitable alternative for older persons because they often prefer to stay in a familiar environment than to move into residential care [1, 2]. Several studies have shown that institutionalization may be associated with adverse outcomes such as depression, lower quality of life, an increase in the use of medication and a rise in mortality [3–8]. Policy makers are therefore keen to foster community care and long-term care policies are shifting to allow older persons to remain in their homes for as long as possible, avoiding early nursing home admission [9–14]. According to a WHO-report (2009), an appropriate balance between care settings for older persons’ care is necessary, including supported self-care and home-based services. The report emphasizes the need for specific interventions to help maintain older people at home and to prevent long-term institutional care [15]. In Belgium, home care interventions are being funded by the National Institute of Health and Disability Insurance, with the purpose of keeping older persons at home with satisfactory quality of life and low informal caregiver’s burden [16]. Scientific evaluation proved their effectiveness in preventing institutionalization [17].

In order to optimize interventions and services in the community, it is important to identify the profile of persons who are able to stay at home and the profile of persons who are being admitted into residential care. Understanding their resources use is also essential as available services may vary according to the care setting. Resources in the context of community care in Belgium are viewed as formal health care (mostly hands-on nursing care at home provided by nurses and skilled help for ADLs provided by nursing assistants) and social care (lower skilled help for IADL, early ADL and chores, as well as social support). The Belgian long term care system can be characterized as a mixed system with extensive and diverse publicly financed formal care services. Care for community dwelling older people is provided by a mix of family practitioners, self-employed nurses, and nurses employed by not-for-profit organizations. Care can also be provided by other care professionals working in an independent practice (i.e. physiotherapists, occupational therapists, psychologists, etc), or employed by for profit or not for-profit private. Practically all services are subsidized [18]. The Belgian health care system is primarily funded through social security contributions and taxation, but there are also out of pocket contributions by patients. The system is based upon the principles of equal access and freedom of choice [19]. Social care and social help are also provided at home and are partially subsidized. Long-term subsidized residential care in Belgium comprises assisted living, care homes and nursing homes.

Eligibility for long-term care in the community and for residential care depends on the degree of care dependency and is evaluated using the 6 items of the Belgian Katz ADL (Activities of Daily Living) scale, adjusted with 2 items about cognitive performance (disorientation in time or space) [20]. Even though this eligibility criterion exists, it is mostly only applied for the funding of residential care. The admission to a nursing home can still be granted to a person with low care dependency and it may still happen that older people are admitted ‘too early’ into residential care, so, at low care levels. This may be the case when the older person has other reasons for not being able to remain at home (loneliness, depressive problems, no social or care network, etc.). The need for an objective tool to identify people at risk of nursing home placement is evident, as health care systems should target the right people in need of residential long term care.

The main objective of the study is to identify persons who are likely to enter residential care based upon their needs and resource utilization. The study describes and compares the case mix of a population of older persons receiving home care services with a group of persons from this population who are being admitted into residential care. By understanding their resource use, care providers can plan interventions effectively and optimize their services and resources to meet the persons’ needs.

## Methods

### Design

This is a longitudinal study including older persons in the community who are at least 65 years old. Participants were enrolled in a larger study called Protocol-3, which evaluated home care interventions in the whole Belgian territory from the year 2010 until 2016, aiming at avoiding early institutionalization. Eligibility criteria were a minimum score of 6 on the Edmonton Frailty Scale or a score indicating moderate ADL-problems in the Katz Scale (Belgian version). Persons with an official dementia diagnosis by a neurologist, psychiatrist or a geriatrician could also participate in the study. All participants signed an informed consent. In case the older person presented cognitive problems or was unable to sign, a family member or a legal guardian signed the document. A research protocol was previously published [16]. Caregivers did not report fatigue of the older persons during assessment completion. The assessment was incorporated into the care practice, is mostly based upon observation and was simultaneously used as a tool for care planning. Home care interventions were classified as follows:

#### Single interventions

1. case management (low intensity, mainly care coordination),
2. occupational therapy (including home adaptations and advice about assistive devices),
3. psychological support (psychosocial support at home),
4. day care (activities and day care support in an institution),
5. night care (offered exclusively to one older person with full supervision during the whole night or to several persons, each with partial supervision),
6. other interventions (not officially specified but they were mostly innovative types of help at home, e.g. assisted living, delivery of medications at home or alternative housing),

#### Multi-component intervention

7. case management with rehabilitation services, occupational therapy and/or psychological support.

The control group consisted of older persons only receiving hands-on nursing care, which is regular home care in Belgium. People in the control group did not receive any intervention, so they can be considered as receiving care as usual.

### Measurements

Professional caregivers received a 2.5-day training in order to fill out the InterRAI Home Care (interRAI HC) instrument. This is an internationally validated comprehensive assessment instrument used in the community setting with the purpose of collecting standardized information about a person’s care needs, clinical situation, functional and cognitive performance, as well as psychosocial and environmental status. The instrument is part of the interRAI Suite of instruments, a standardized set of comprehensive assessments for different care settings with a validated common core of items, which can provide tools for care planning and can improve continuity of care, by information transfer across settings and by enhancing multi-disciplinary work [21]. A joint report of the OECD and the European Commission (2013) showed that the interRAI instruments enable integrated eCare and allow high quality data sharing between organizations and settings [22]. Professional caregivers assessed participants at baseline (at inclusion in the program) and every six-months until the older persons were admitted into residential care, stopped receiving the intervention or died. If older people were admitted into residential care in the period between assessments, a new assessment was required in order to evaluate the situation of the older person at admission. The follow-up period could then be at a different time than six months or one year, in case the older person was admitted before or after six months. The analysis included the interRAI-HC assessments at baseline (community care) and at follow-up (in community care or at admission to residential care).

### Outcome

The main outcome variable in the study is the Case-Mix Index derived from the interRAI Resource Utilization Group (RUG)-III system. The interRAI RUG-III system classifies persons into a total of 34–44 resource utilization groups (RUGs), with a corresponding Case-Mix Index (CMI) for each of these RUG groups. The case-mix indexes reflect the relative ‘amount’ of resources necessary for an older person in a certain RUG-III group compared to an older person in another RUG-III group. It is a measure of the amount of time (‘cost’) spent in care. Persons in a group of high level of resources utilization will have a higher CMI than an older person in a group with low resource intensity. The RUG-III system was validated and is used internationally for classification of direct care resource use and for management purposes (e.g., determining staff levels) in residential care. It is calculated with the items from the interRAI Long-Term Care Facilities assessment (interRAI-LTCF) [23–28]. The analogous RUG-III/Home Care (RUG-III/HC), which was used in this research and derived from the interRAI Home Care (interRAI-HC) assessment, is the validated version for the community setting [29, 30]. The RUG-III/HC is constructed upon more than 60 items from the interRAI HC instrument.

There are seven hierarchical categories in the RUG-III/HC system: Rehabilitation, Extensive Services, Special Care, Clinically Complex, Impaired Cognition, Behavior Problems, and Reduced Physical Functioning. These seven hierarchical categories are further differentiated into 23 groups according to the resources used (e.g. minutes rehabilitation – speech therapy, physiotherapy or occupational therapy; extensive services, special care or clinically complex care) or health and functional situation (impaired cognition, behavior problems or physical impairment). The secondary split is based on an Activities of Daily Living index (ADL-index) with 4 ADL items (toilet transfer, toilet use, mobility in bed and eating) and this split further differentiates within these groups. Higher scores on the ADL-index point to higher ADL impairment. The tertiary split divides some groups even further based upon the score on the Instrumental Activities of Daily Living Performance scale (IADLP) scores. Similarly, higher scores on IADLP indicate higher IADL impairment.

### Analysis

Data analysis was performed in two steps using SAS-9.4. First, descriptive statistics were calculated describing the characteristics and the health status of older persons in the community (baseline). Subsequently, the RUG-III/HC algorithm was applied for all persons in the community (at baseline) and at follow-up. From the RUG-III/HC, the CMI for each person was calculated, which is the main outcome of the study.

To compare differences between the CMIs for both populations, the Mann-Whitney U test was used, as this test can detect differences in spread even when the medians are very similar [31]. Proportion tests were used for some interRAI outcomes and items. Additional outcomes used were the interRAI Activities of Daily Living Hierarchy scale (ADLH) [32], the Instrumental Activities of Daily Living Performance scale (IADLP) [33], the Cognitive Performance scale [34] (CPS version 2) and the Depression Rating scale (DRS) [35]. For the analysis in this research, the interRAI scales were dichotomized according to their internationally validated cut-offs: ADLH (range 0–6, score ≥ 3 indicating extensive assistance for ADL), IADLP (range 0–48; score ≥ 24 indicating extensive needs for IADL); CPS2 (range 0–6; score ≥ 3 indicating moderate to severe cognitive impairment); DRS (range 0–14; score ≥ 3 indicating presence of depressive symptoms). The use of the interRAI scales can be justified by the fact that they have been internationally validated based on “gold standard” measures [36, 37]. To analyze the change between the CMI scores at baseline and at follow-up, a linear multivariable regression model was constructed with the covariates at baseline for age, gender and the interRAI scales ADLH, IADLP, CPS2 and DRS. The value of the CMI at baseline was included in the model to control for baseline values. Multi-collinearity tests were performed prior to the inclusion of the covariates in the regression.

## Results

### Characteristics of the study population

The study population consisted of 10,289 older persons living in the community (81.2 ± 7.1 yrs., 69.1% female). A total of 9692 people were in the intervention group and 597 people were in the control group. The mean follow-up time in the study was 353 days and the median was 185 days. Most older people were living alone (55.3%) and 82.2% had at least one informal caregiver, with a total of 39.5% of the study population living together with their informal caregivers. Table 1 shows the characteristics of the population at baseline. The majority of older people (78.2%) needed at least extensive assistance in IADL (score > =24) and 49.9% in ADL (score > =3). A total of 28.1% of the participants were at least moderately cognitively impaired (score > =3). Moreover, 27.6% of the older persons presented daily depressive symptoms. Urinary incontinence was frequent (28.5%) as well as the incidence of falls in the last 90 days (38.5%). From this population, 853 persons (8.3%), mostly female (83.7%) left the community into residential care (average age: 83.7 yrs.).

**Table 1:** Baseline characteristics of the study population

Baseline Characteristics	N = 10,289 / Age (±SD): 81.24 ± 7.06 yrs. % [C.I.] †
Gender: female	69.14% [68.24; 70.03]
Living alone	55.32% [54.36; 56.29]
Availability of an informal caregiver	82.28% [81.53; 83.02]
Living with the informal caregiver	39.50% [38.73; 41.45]
IADL Performance scale (range 0–48) value ≥24 Median	78.18% [77.34; 79.02] 34
ADLH scale (range 0–6) value ≥3 Median	49.89% [48.92, 50.86] 2
CPS2 scale (range 0–6) value ≥3 Median	28.12% [27.25, 28.99] 1
DRS scale (range 0–14) value ≥3 Median	27.60% [26.73, 28.47] 1
Incidence of falls in last 90 days	38.49% [37.55, 39.44]
Urinary incontinence	28.52% [27.65, 29.39]

† [95 *C.I.*] = 95% confidence interval] Instrumental Activities of Daily Living Performance (IADLP) Activities of Daily Living Hierarchy (ADLH) Cognitive Performance-2 (CPS-2)

Depression Rating Scale (DRS) Table 2: Average CMI per type of intervention

### Resource utilization of the study population

**Fig. 1:**
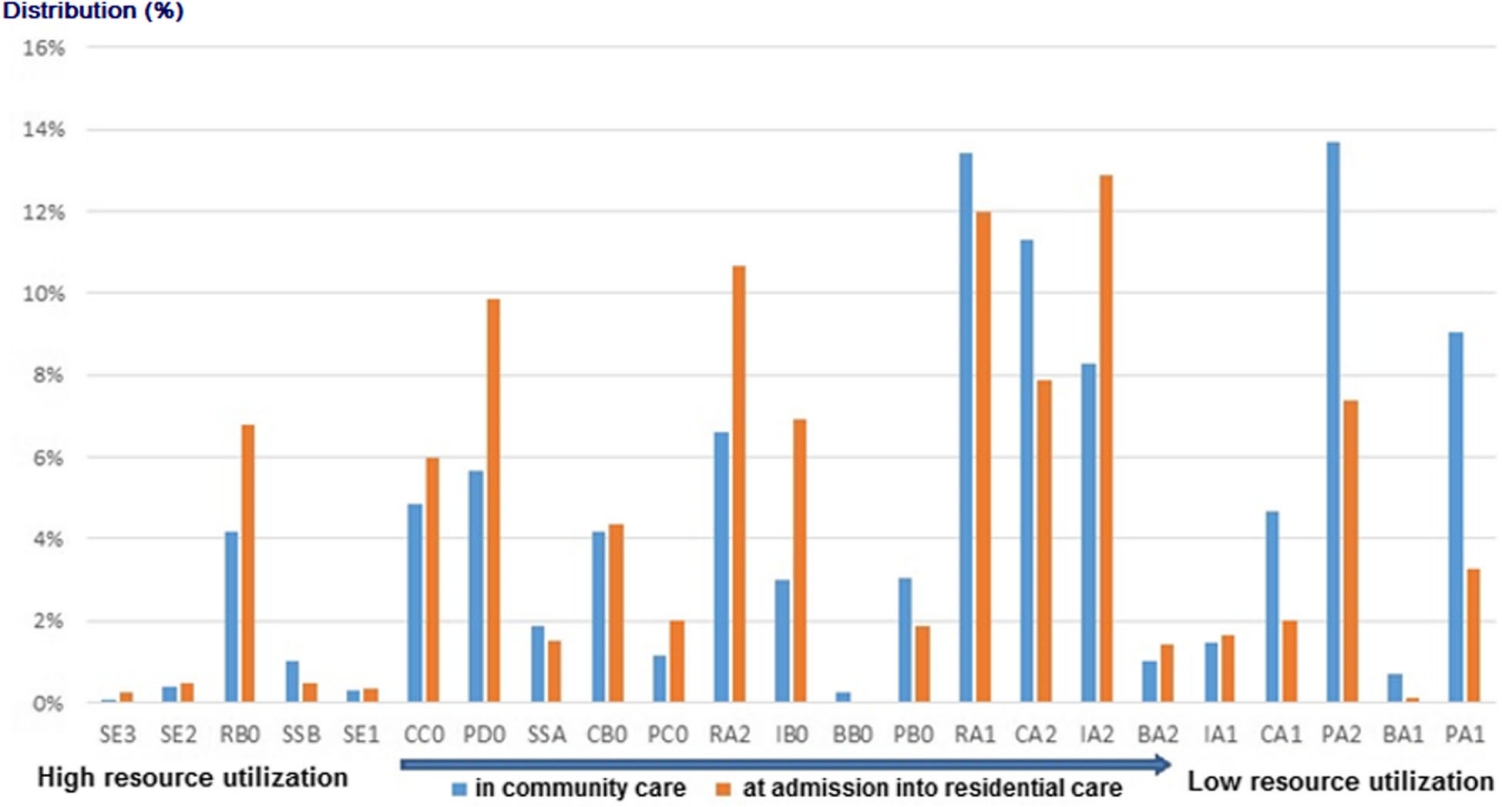
Comparison of the RUG-III/HC distribution of older persons in the community and at nursing home admission ordered by Case Mix index (CMI)

The distribution of older persons based on the RUG-III/HC groups is shown in Fig. 1. The RUG-III/HC groups were ordered by CMIs, with the groups with the highest resource intensity on the left of the X-axis. The RUG-III/HC group PA1 (Physical Functioning ADL–low levels of ADL and IADL-impairment) was the group with the lowest resource intensity and the highest was SE3 (Extensive Special Care 3). The largest proportion of the community setting population was found in the PA2 (Physical Functioning ADL–low level of ADL-impairment, high level of IADL-impairment) group (13.7%). These are persons with neither major ADL-dependencies (in toilet transfer, toilet use, mobility in bed or eating), nor complex nursing care needs, but who need assistance in IADL (meal preparation, managing medications and telephone use). The second most populated RUG-III/HC group was RA1 (Low Rehabilitation, with low ADL and low IADL- impairment – 13.4%), followed by CA2 (Clinical Complex– 11.3%) and PA1 (Physical Functioning ADL– low levels of ADL and IADL-impairment– 9.1%). Among the participants being admitted to residential care, the largest group was the Cognitive impairment IA2 (Cognitive Impairment with low ADL but high IADL-impairment) with a distribution of 12.9%. The second most populated group at the entry point into residential care was RA1 (Low Rehabilitation, with low ADL and low IADL-impairment–12.0%), followed by 10.7% in RA2 (Rehabilitation Medium with low ADL-impairment but high IADL-impairment) and 9.9% in PD0 (Physical Functioning ADL– high level of ADL-impairment).

**Fig. 2:**
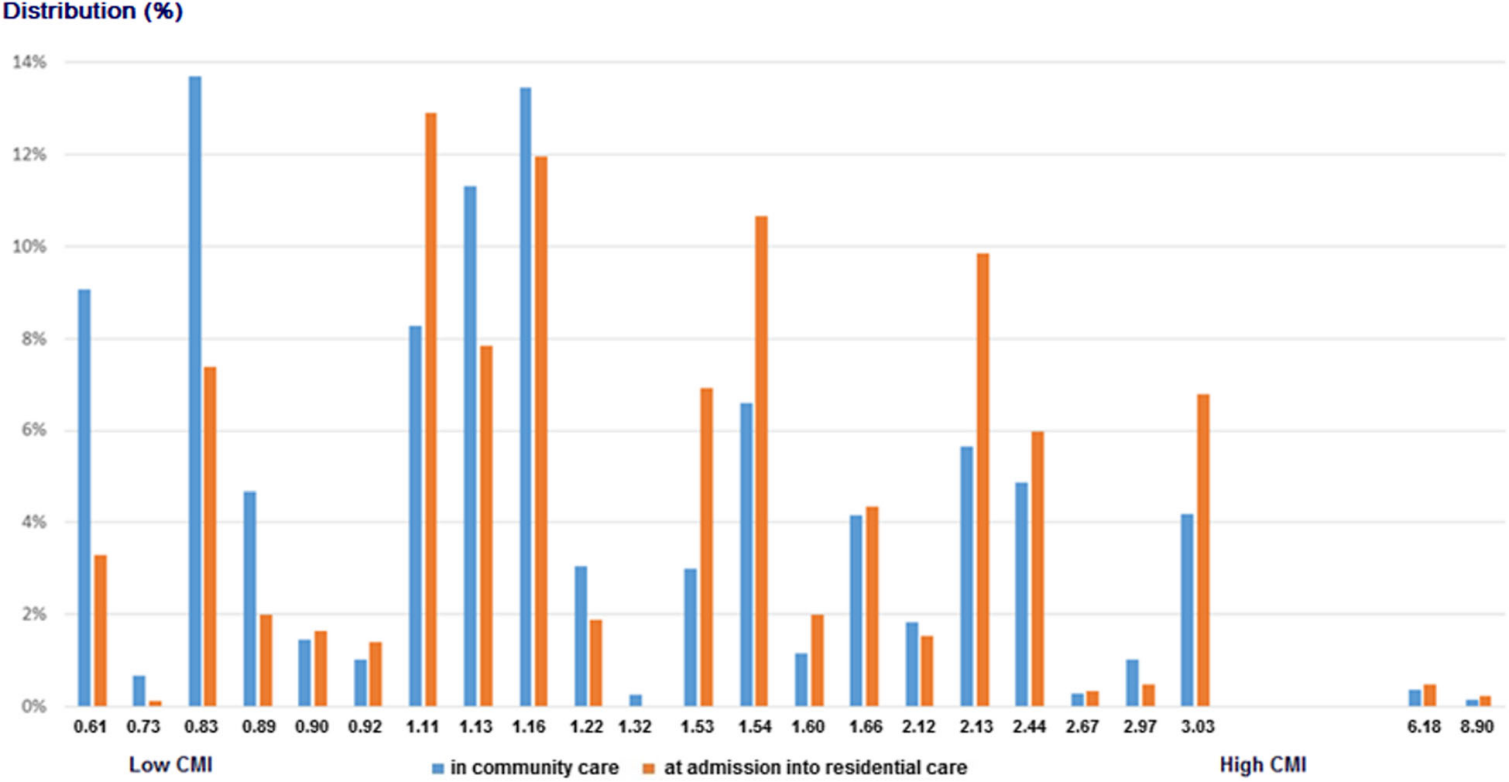
Comparison of the RUG-III/HC case-mix index (CMI) distribution of older persons in the community (at baseline) and at nursing home admission (follow-up)

Figure 2 shows the distribution of the CMIs for formal care for the whole study population. As an example, a person in a group of rehabilitation (RB0), with a CMI of 3.0, uses approximately 4.9 times more resources than a person in the lowest resource group (PA1), with a CMI of 0.6. As the average in the sample of community care persons was 1.4, a person in a sub-group of PA1 (CMI 0.4) used 45% of the resources of an average person in the sample. Additionally, the average value for the CMI for the persons entering residential care was 1.6. As expected, the average value of the CMI for this population was significantly higher than the value for the population in community care (*p* ≤ .001).

In both settings, there were persons with high and low resource use, but in different proportions. Among participants who were being admitted to residential care in low resource utilization groups (Physical Function PA: PA1 and PA2 together), 41.8% needed assistance for some ADLs (e.g. personal hygiene and dressing), 79.5% had at least extensive assistance needed for IADL (IADLPscore> = 24), and 31.9% presented mild cognitive problems (CPSscore = 2). Additionally, 37.8% of the persons in the PA group had daily depressive symptoms (DRS score > =3) and 36.4% felt lonely (self-report item ‘feels lonely’ from the interRAI-HC). Therefore, even if these persons were in the lowest RUG-III/HC group, they did need assistance in daily activities and psychosocial support, or felt the need to be in an environment with other persons. In the community care, in the lowest RUG-III/HC group Physical Function PA (grouping PA1 and PA2 together), 19.9% had ADL needs (mostly help for personal hygiene and dressing) and 39.8% had needs for IADL. In this group, 15.7% of the persons presented mild cognitive impairment (CPSscore = 2) and 25.5% of the persons had daily depressive symptoms (DRSscore> = 3) or reported that they felt lonely at home (36.4%. This means that persons in the community grouped into the lowest category (Physical Function PA) would also need formal help if informal support is unavailable or insufficient.

For the persons in community care who had high resource utilization, since they were still living at home, the needed resources were provided at home, ambulatory or during a short stay in a nearby residential setting. In the community, we also found some persons with very high resource utilization such as SE3 (Extensive Special Care-3) and High Complex Care with high ADL-impairment (CC0).

### Interventions and resource utilization

The average CMIs for each type of home care intervention and the control group are presented in Table 2. The CMIs of the participants receiving night care were the highest (1.6) followed by persons receiving occupational therapy (1.5) and persons enrolled in case management interventions with rehabilitation, occupational therapy combined or not with psychological support (1.4). Persons in these three types of interventions had higher resource utilization because the RUG system placed persons in higher groups according to their use of services and therapies. Persons in the control group had an average CMI of 1.4. People with the lowest CMIs were receiving case management with low-intensity (1.3), psychological support (1.0) or other types of interventions (1.2). Only persons receiving night care had a similar CMI of people at admission into residential care (1.6). In the groups of persons receiving case management solo, case management with rehabilitation (with or without psychological support) or receiving night care, the CMI of persons being admitted to a residential setting was significantly higher than the CMI at baseline. Thus at the admission into residential care, their resource utilization was significantly higher.

**Table 2:** Average CMI per type of intervention at baseline and follow-up

Type of community care intervention	Baseline: Total population in community care (*N* = 10,289) Average CMI: 1.36 ± 0.69	Follow-up: Population admitted to residential care (*N* = 853) Average CMI: 1.57 ± 0.79 ***	
	Average CMI ± SD	Average CMI ± SD	*p*-value
Case management with rehabilitation, occupational therapy and/or psychological support	1.35 ± 0.63	1.56 ± 0.82	*p* = 0.000 ***
Case management only	1.28 ± 0.69	1.56 ± 0.80	p = 0.000 ***
Day care	1.34 ± 0.66	1.49 ± 0.72	*p* = 0.54
Night care	1.57 ± 0.91	1.70 ± 0.63	*p* = 0.001 ***
Occupational therapy	1.47 ± 0.85	1.64 ± 0.58	*p* = 0.11
Psychological support	1.04 ± 0.43	1.29 ± 0.43	*p* = 0.92
Other interventions (alternative housing, medication delivery, etc.)	1.18 ± 0.58	1.38 ± 0.39	*p* = 0.66
Frail home care population receiving only nursing care (control group)	1.42 ± 0.77	1.38 ± 0.55	*p* = 0.87

*** *p* ≤ 0.001

### CMI change between baseline and follow-up

Table 3 shows the results of the multivariable regression model for the CMIs at follow-up controlling for the value of the CMI at baseline and the covariates age, gender, ADLH and IADLP scores, CPS2 score and DRS score at baseline. Tests showed no multi-collinearity among covariates. The model yielded significant coefficients for all variables, except for age. As expected, the CMI value at baseline showed the highest coefficient (0.61). Being female showed an inverse effect on the CMI at follow-up. The interRAI scales are all significant covariates and yielded small, but positive coefficients. The coefficient for being admitted to residential care at follow-up was positive and significant (0.12), indicating that persons at nursing home admission presented higher CMI values than people remaining in the community. A model for the difference between the CMI at follow-up and the CMI at baseline as the dependent variable yielded similar results, but is not presented here.

**Table 3:** Multivariable regression model for CMI at follow-up

CMI at follow-up Mean follow-up time: 353 days Median follow-up time: 185 days	Coefficient	p-value	[C.I.] †
Age at baseline	−.001	0.263	[−0.003; 0.001]
Gender: female	−.038	0.003**	[−0.062; − 0.013]
CMI at baseline	.607	0.000***	[0.584; 0.629]
Admitted to residential care at follow-up (yes = 1)	.123	0.000***	[0.085; 0.162]
CPS2 score at baseline	.035	0.000***	[0.028; 0.043]
ADLH score at baseline	.023	0.000***	[0.014; 0.033]
IADLP score at baseline	.006	0.000***	[0.005; 0.007]
DRS score at baseline	.007	0.003**	[0.002; 0.012]

R-squared: 0.4979 † [95 *C.I.*] = 95% confidence interval] CMI: Case-mix Index

Instrumental Activities of Daily Living Performance (IADLP) Activities of Daily Living Hierarchy (ADLH)

Cognitive Performance-2 (CPS-2) Depression Rating Scale (DRS)

***p ≤ 0.000 **p ≤ 0.01

## Discussion

This longitudinal study compared the case mix of a population of older persons in the community with a group of persons from this population who were being admitted into residential care at follow-up. In both settings, there was a large variability across the RUG categories with people in low, medium and high intense groups. This finding is congruent with Shugarman et al. (1999), where the case-mix of nursing home residents in Ohio was compared with the case-mix of persons receiving community care in Michigan, using a previous version of the interRAI instruments - the RAI-2.0 [23]. The studyshowed some overlap of the RUG-III categories across the samples from Ohio and Michigan, but also showed a large variability across RUG groups. Similarly to our study, persons who were very resource intense were also cared for in the community setting (CC0 group) and persons who were only minimally resource intense were also found in residential setting (PA: Reduced physical function ADL).

We could argue why participants in the lowest group of resource utilization (PA) were targeted by the interventions or were being admitted into residential care. Our study showed however that many of the older persons in the PA group still had moderate ADL-impairment and often needs for IADL-assistance as well as mild cognitive impairment. As they were also at risk of institutionalization, interventions designed to avoid early nursing home admission should target persons with similar levels of impairment as well [38, 39]. In addition, persons in the lowest levels of ADL, IADL and cognitive impairment could still be in need of help because of loneliness or depressive symptoms.. Engaging them in activities and offering psychological support and guidance would be advisable [17]. Supervision and help for persons with mild cognitive problems or psychological support for persons with depression can still require substantial resources. In the case of depression, the support needed cannot be accounted for in the RUG-III/HC since the depression scale (DRS) is not included in its algorithm, neither any item about psychotherapies. The DRS scale is only included in the RUG-III for residential care.

A joint report from the OECD and the European Commission (2013), which presented the RUG-III distribution of people in the community and residential settings with samples from nine countries, also showed an overlap across RUG-III groups and settings [22]. Except for the RUG-III/HC group Extensive Services, all groups had persons in both settings. Similarly, in our sample, there were almost no persons in the highest resource levels (no participants in Rehabilitation High or Very High and a few people in Extensive Care 3). This finding is consistent with previous studies [22, 23] since very high levels of intensive care tend to be available only in specialized skilled units in residential care.

The advantage of using resource utilization groups was confirmed by our study and previous research [40, 41]. Our findings showed additional possibilities of the RUG-III/HC system to identify persons based upon their CMIs and for allocating resources to keep older people at home longer. The results showed the importance of the availability of rehabilitation services in the community setting, allowing older people to follow rehabilitation therapies at home. If these therapies were not available at home or in short-term rehabilitation centers, older persons needing rehabilitation could be institutionalized too early. The results were in line with the results of previous research [17], which proved the effectiveness of interventions offering rehabilitation to older persons in the community in order to avoid early institutionalization. In addition, persons receiving night care presented the highest relative risk of being admitted into residential care, which was also conclusive with our results considering the CMI distribution of persons receiving night care (highest CMI at baseline: 1.57 ± 0.91). Their average CMI in the community was as high as the average CMI of persons being institutionalized (1.57). They correspond to people with the highest CMIs in the community and keeping them at home is not always possible or desirable. The services they received at home were at high level of resource utilization, as they needed assistance during the day and night, which is not always feasible in the community setting. Older people with high levels of CMI tend to move into residential care in the near future. Providing high level of care at home is not always optimal and moving into residential care may be considered a cost effective solution [42, 43]. In addition, our findings showed that the CMI values at follow-up for persons being admitted to residential care was higher than the CMI values for persons remaining at home. Although in the constructed linear model, the proportion of variance in the dependent variable that could be explained by the independent variables was 49% (R-squared< 0.5), some fields of study have inherently greater amount of unexplainable variation. We believe that some variables may be lacking, like choice of going into residential care, social factors, family wishes, etc. Many studies trying to explain human behavior have generally R-squared values lower than 50% [44]. Nevertheless, we could still draw important conclusions from the model, as many relevant independent variables showed to be statistically significant. The model indicated the possibility of using the RUG-III system for eligibility to residential care. As the RUG-system takes into account the needs as well as the level of utilization of some therapies, caregivers have to be aware whether persons are receiving all the services they need. Underutilization of services due to lack of access to care (e.g., because of unavailability of services or financial issues) are very relevant in this case. If an older person is placed in a high ADL need group (PE), but also should be receiving high level of rehabilitation, this older person will be classified into a lower RUG group than people who have the same level of ADL needs and already receive rehabilitation services (as the CMI of the PE group is lower than RC). It is the responsibility of policy makers and caregivers to target persons with unmet needs and to organize and offer the services and therapies they need. Offering the necessary therapies can help older people to stay at home longer, as unavailability of home care services can often be a reason for nursing home placement [45]. Literature shows that countries that succeed to provide effective community-based care and services are likely to optimize their public spending [46].

Moreover, if informal caregivers are the main carers, persons often will use a lower level of formal care or therapies, such as nursing care or occupational therapy. Therefore, persons receiving informal care instead of formal care will also be in a lower RUG group, in spite of the fact that they do use resources (time) from informal care. For this reason, informal care time should also be included in the calculation of the RUGs. The RUG system can account for some of the time informal caregivers spend, but not for all. A particular innovation in RUG-III/HC (23 groups) was that informal care time was used in its validation and an additional set of CMIs was calculated including informal care time. In this case, especially emotional support and supervision offered by informal caregivers may be still underestimated. In our study, we could not account for any informal care time.

### Strengths and limitations

The main limitation of the research was the fact that the study population was not representative of all older persons living in the community, as the participants enrolled in the study were receiving home care services. Another limitation is the lack of information about the reason for admission into nursing home and we cannot explain the main reasons why some people with very low resource utilization moved into residential care. A mixed methods approach could help us identify these reasons, which may be social, emotional, etc. Strengths of the research were the large longitudinal study population and the use of the interRAI-HC, as it contains substantial information about the older persons’ situation, enabling a multifactorial approach. This study recognizes the significant contribution of informal care towards care at home, however informal care time was beyond the scope of this study.

## Conclusion

The study showed the differences between the resource utilization of persons receiving community care and persons being admitted into residential care. Results show that the RUG-III/HC offers possibilities for identifying persons at risk of institutionalization in the community, based upon their case-mix index. Based on the case-mix, resources can be allocated to keep older persons at home longer, bearing in mind the complexity of care and the availability of services in the community. Interventions designed to avoid early institutionalization should use tools like the interRAI-HC and its outcome variables like the RUG-III/HC, which are suitable for care planning and for allocation of services, in order to optimize care and the use of resources.

## Data Availability

The data that support the findings of this study are available from Healthdata.be but restrictions apply to the availability of these data, which were used under license for the current study, and so are not publicly available. Data are however available from Healthdata.be upon reasonable request and with permission of the Belgian Privacy Commission.

## Abbreviations

ADL: Activities of Daily Living
IADL: Instrumental Activities of Daily Living
interRAI RUG-III: InterRAI Resource Utilization Groups III
RUG-III/HC: InterRAI Resource Utilization Groups III for the Home Care ssetting
interRAI HC: InterRAI Home Care
interRAI LTCF: InterRAI Long Term Care Facilities
CMI: Case-Mix Index
IADLP: Instrumental Activities of Daily Living Performance Scale
ADLH: Activities of Daily Living Hierarchy Scale
CPS: Cognitive Performance Scale
DRS: Depression Rating Scale
